# Making the 2022 Rwanda Census count.

**DOI:** 10.23889/ijpds.v7i3.1846

**Published:** 2022-08-25

**Authors:** Rachel Shipsey

**Affiliations:** 1 Office for National Statistics

For the 2022 Rwanda Census, data will be collected electronically for the first time. This provides both an opportunity and a challenge to use technology for census processing. We are working with National Institute of Statistics Rwanda (NISR) to help overcome linkage problems and produce robust population estimates.

Working with NISR we aim to create an achievable Census solution given the constraints on time, computing power and experience. We identified key issues, such as the lack of addresses and full date of birth, and developed a series of recommendations. These combined our experience of census processing and NISR’s knowledge of Rwandan geography, conventions and culture to develop the simplest solutions possible, ensuring that each recommendation gave maximum benefit for the effort required. Next, we plan to use 2021 Rwanda Census Pilot data to begin development of an end-to-end matching exercise including pre-processing, automatic linkage, clerical resolution and quality assurance.

We developed recommendations, including small changes to the data collection application and creating a Unique Property Reference Number by concatenating the Enumeration Area code and the Structure Number written on the building during Census collection. Following this, we produced a synthetic Rwandan Census and Post Enumeration Survey (PES) which was used to demonstrate and collaboratively develop matching algorithms. Probabilistic linkage methods were considered but discarded due to lack of sufficient infrastructure. Instead, deterministic methods were used. Match keys are currently being developed by NISR with clerical review undertaken using our open-source Clerical Matching Online Widget (CROW) with the synthetic data. Future plans include using the Census Pilot to estimate overcount and developing a modified version of the Soundex phonetic encoder for use on Rwandan names.

The resource available for the Rwandan Census is comparatively small, but even the smallest resource can have a massive impact when used productively. This collaborative project takes the key concepts of well-developed linkage strategy and makes appropriate modifications to enable an achievable and effective Rwandan Census linkage strategy.

